# Socio-Cultural Disparities in GDM Burden Differ by Maternal Age at First Delivery

**DOI:** 10.1371/journal.pone.0117085

**Published:** 2015-02-13

**Authors:** Marion Abouzeid, Vincent L. Versace, Edward D. Janus, Mary-Ann Davey, Benjamin Philpot, Jeremy Oats, James A. Dunbar

**Affiliations:** 1 Greater Green Triangle University Department of Rural Health, Flinders and Deakin Universities, Warrnambool, Victoria, Australia; 2 Department of Epidemiology and Preventive Medicine, Monash University, Victoria, Australia; 3 Department of Medicine, Northwest Academic Centre, The University of Melbourne and Western Health, Victoria, Australia; 4 Consultative Council on Obstetric and Paediatric Mortality and Morbidity, Clinical Councils Unit, Department of Health, Victoria, Australia; 5 Mother and Child Health Research, La Trobe University, Victoria, Australia; 6 Melbourne School of Population and Global Health, The University of Melbourne, Victoria, Australia; National Research Council (CNR), ITALY

## Abstract

**Aims:**

Several socio-cultural and biomedical risk factors for gestational diabetes mellitus (GDM) are modifiable. However, few studies globally have examined socio-cultural associations. To eliminate confounding of increased risk of diabetes in subsequent pregnancies, elucidating socio-cultural associations requires examination only of first pregnancies.

**Methods:**

Data for all women who delivered their first child in Victoria, Australia between 1999 and 2008 were extracted from the Victorian Perinatal Data Collection. Crude and adjusted GDM rates were calculated. Multivariate logistic regression was used to examine odds of GDM within and between socio-cultural groups.

**Results:**

From 1999 to 2008, 269,682 women delivered their first child in Victoria. GDM complicated 11,763 (4.4%) pregnancies and burden increased with maternal age, from 2.1% among women aged below 25 years at delivery to 7.0% among those aged 35 years or more. Among younger women, GDM rates were relatively stable across socioeconomic levels. Amongst older women GDM rates were highest in those living in most deprived areas, with a strong social gradient. Asian-born mothers had highest GDM rates. All migrant groups except women born in North-West Europe had higher odds of GDM than Australian-born non-Indigenous women. In all ethnic groups, these differences were not pronounced among younger mothers, but became increasingly apparent amongst older women.

**Conclusions:**

Socio-cultural disparities in GDM burden differ by maternal age at first delivery. Socio-cultural gradients were not evident among younger women. Health and social programs should seek to reduce the risk amongst all older women to that of the least deprived older mothers.

## INTRODUCTION

Gestational diabetes mellitus (GDM) begets diabetes. Following a GDM pregnancy, recurrence rates in subsequent pregnancies range from 30% to 84% [1]. GDM also increases the risk of development of type 2 diabetes in both mother [2] and infant [3].

For many conditions and in many settings, associations of socioeconomic forces with health status are increasingly being described. Despite recognition of the associations of social factors with deranged glucose metabolism and its sequelae, there has been little attention to the socio-cultural factors associated with GDM [4–14]. Some aspects of reproductive history that are associated with GDM vary between socio-cultural groups. For example, family size and parity may differ between women from different socio-cultural backgrounds and this may differentially influence GDM risk due to issues of GDM recurrence and effects of inter-pregnancy weight gain. When examining socio-economic associations with GDM, considering only women giving birth for the first time may remove the potential for confounding by some such factors and for residual confounding by reproductive influences that cannot easily be measured.

GDM is associated with a range of potential adverse outcomes [15]. Several risk factors for GDM are potentially modifiable. So too are many of the social determinants of health, and it is important that deprivation does not beget deprivation that begets diabetes. From an epidemiological and equity perspective, given that the intrauterine environment influences an infant’s future disease risk, identifying and addressing any socio-cultural differences in GDM burden may help reduce intergenerational health inequities and modulate future socio-cultural disease gradients.

We examined the rates of GDM among primiparous women who delivered in the state of Victoria, Australia over a ten-year period and the associations between socio-cultural factors and GDM burden.

## METHODS

Under Victoria’s public health legislation, all births occurring in the state that are over 20 weeks’ gestation or if birthweight is unknown, those with a birthweight greater than 400g, are notified to the Victorian Perinatal Data Collection (VPDC). Birth report notification forms are completed by clinicians. Information collected includes maternal demographic characteristics, reproductive history, and details of ante-, peri- and post-partum progress and complications.

De-identified data for all births to primiparous women who delivered in Victoria between 1 January 1999 and 31 December 2008 were extracted from the VPDC. Primiparous women are those giving birth for the first time. For multiple gestations (i.e. twins, triplets etc.), only the birth record for the first-born infant was included; each record therefore denotes one pregnant woman. Datafields obtained for use in this analysis were maternal age at delivery (categorised as < = 24 years, 25–29, 30–34, > = 35 years), maternal country of birth, maternal Aboriginal and Torres Strait Islander status, residential area postcode, marital status at the time of delivery (married/de facto or single/separated/divorced/widowed), birth plurality (singleton or multiple gestation) and GDM status in the index pregnancy (yes/no). GDM status was assigned based on whether the ‘gestational diabetes’ box on the birth report form was ticked by the clinician notifying the birth; accuracy of reporting of GDM in the VPDC is near complete [16] and screening and diagnostic criteria for GDM in Australia were consistent over the study period [17]. We did not consider gravidity, and so did not exclude women who gave birth to their first child but may have had a previous pregnancy that was not carried to 20 weeks gestation.

Maternal country of birth was reclassified into geographic regions, using the Australian Bureau of Statistics’ *Standard Australian Classification of Countries*. We recategorised the ‘Oceania and Antarctica’ group used in this classification system into three distinct categories: *Australia (non-Indigenous)*, *Australia (Indigenous)*, and *Oceania*. Residential area postcode at the time of delivery was used to assign a residential area socio-economic status (SES) score, using the Australian Bureau of Statistics’ *Index of Relative Socio-Economic Disadvantage* (IRSD). This 17-item composite measure incorporates area-based markers of socio-economic disadvantage, such as the proportion of residents who are unemployed, on low incomes, have low educational attainment, lack access to a car and so on [18]. Low scores represent relatively high levels of disadvantage in an area, whereas high scores represent a relative lack of disadvantage. In this study, each woman was assigned an IRSD score according to that of her residential postal area. SES quartiles were then generated by dividing women into four groups, such that quartile 1 comprised the 25% of women living in the most deprived areas and quartile 4 contained the 25% of women living in the least deprived areas. IRSD scores used to define quartiles were 765-, 969–970, 1017–1018 and 1055–1056. These values generally approximated those obtained if quartiles were generated based on postcodes, with quartile 1 containing the most deprived postcodes and so on.

### Statistical analysis

Data for the ten-year period were analysed collectively, using Stata 12.0. Descriptive statistics were used to profile demographic characteristics of all primiparous women and those whose pregnancies were complicated by GDM. Characteristics of women with and without GDM were compared using t-tests for continuous measures and chi-square tests for categorical variables. Crude GDM rates were calculated using the proportions function, and logistic regression and the margins command yielded adjusted incidence rates. Logistic regression was used to examine univariate associations between GDM and each variable. Multivariate logistic regression was used to compare odds of GDM by SES, adjusting for maternal age group and additionally, each of maternal region of birth, marital status and plurality. Similar regression analyses were used to examine associations between GDM and region of birth, using maternal age group and each of SES, marital status and plurality as explanatory variables.

The Consultative Council on Obstetric and Paediatric Mortality and Morbidity, Victorian Department of Health, granted permission to access and analyse the data. The Flinders University Social and Behavioural Research Ethics Committee exempted this study from ethics approval as it involved analysis of existing de-identified data.

## RESULTS

### Demographic characteristics of all primiparous women who gave birth in Victoria between 1999 and 2008

In the decade to December 2008, 269,682 women gave birth to their first child in Victoria and were registered with the VPDC. All but 922 (0.3%) could be assigned to an SES group and region of birth was known for all but 1153 (0.4%). Women with pre-existing diabetes comprised 0.4% of all primiparous women who delivered during this period; these women were retained in the denominator but not considered separately.

Demographic characteristics varied by SES (Table 1). Overall, women living in the most deprived areas were on average younger than women residing in less deprived areas. Those in the most deprived areas also had lower rates of multiple gestation pregnancies and were less likely to have a partner (data not shown). Demographic characteristics also varied by maternal region of birth (Table 1).

**Table 1:** Demographic characteristics of primiparous women delivering in Victoria by residential area socioeconomic status* and maternal region of birth, Victoria 1999–2008.

RESIDENTIAL AREA SES *	*n* (%)		Maternal age at delivery Mean (SD)		SES Mean (SD)	
	All women	GDM	All women	GDM	All women	GDM
1 *(most deprived)*	62578 (23.2)	3040 (25.8)	26.9 (5.7)	29.9 (5.3)	918 (40)	911 (41)
2	66934 (24.8)	2800 (23.8)	27.9 (5.5)	30.1 (5.2)	993 (13)	993 (12)
3	67120 (24.9)	2913 (24.8)	29.1 (5.1)	31.0 (4.9)	1034 (11)	1034 (11)
4 *(least deprived)*	72128 (26.7)	2975 (25.3)	30.8 (4.8)	32.0 (4.6)	1081 (20)	1080 (19)
REGION OF BIRTH						
Australia *(non-Indigenous)*	202374 (75.0)	6970 (59.3)	28.7 (5.5)	30.5 (5.1)	1013 (59)	1012 (60)
Oceania	6197 (2.3)	267 (2.3)	28.4 (6.1)	30.8 (5.7)	1007 (67)	1000 (69)
North-West Europe	9352 (3.5)	371 (3.2)	31.6 (4.8)	33.1 (4.6)	1033 (54)	1032 (54)
Southern & Eastern Europe	6443 (2.4)	371 (3.2)	29.4 (5.5)	31.8 (5.4)	997 (73)	995 (72)
North Africa & Middle East	5204 (1.9)	266 (2.3)	26.0 (5.6)	29.6 (6.0)	956 (82)	951 (85)
South-East Asia	15585 (5.8)	1438 (12.2)	28.8 (5.0)	31.0 (4.9)	976 (81)	974 (80)
North-East Asia	6573 (2.4)	733 (6.2)	30.7 (4.6)	32.5 (4.3)	1023 (66)	1021 (66)
Southern & Central Asia	8435 (3.1)	903 (7.7)	28.1 (4.4)	29.9 (4.3)	993 (72)	989 (72)
Americas	3322 (1.2)	150 (1.3)	30.2 (5.3)	32.0 (4.9)	1020 (67)	1017 (64)
Sub-Saharan Africa	3489 (1.3)	206 (1.8)	28.9 (5.2)	30.3 (4.6)	1005 (72)	988 (73)
Australia (Indigenous)	1555 (0.6)	41 (0.3)	22.6 (5.9)	25.9 (6.0)	966 (58)	991 (58)
Region unknown	1153 (0.4)	47 (0.4)	27.1 (5.5)	29.2 (4.8)	983 (74)	975 (61)
OVERALL	269682 (100)	11763 (100)	28.7 (5.5)	30.8 (5.1)	1009 (64)	1004 (67)

Note that column totals will vary as they exclude women for whom the variable of interest is not known *Excludes 922 women for whom residential area SES quartile not known.

### Demographic characteristics of women whose pregnancies were complicated by GDM

GDM complicated 11,763 pregnancies (4.4%). Women who developed GDM were on average somewhat older than those without GDM (30.8 years vs. 28.7 years) and resided in areas of lower SES (mean IRSD score 1004 vs. 1010); significantly more of those with GDM had a multiple pregnancy (2.8% vs. 1.9%, p<0.001 for all).

GDM rates increased with rising maternal age, from 2.1% among women aged below 25 years at delivery to 7.0% among those aged 35 years or more. GDM was less likely in women without a partner compared with women who were married or de facto (11.5% vs. 16.7%, OR 0.65, 95% CI 0.61–0.69). Odds of GDM were 1.52 times higher in women with multiple gestation compared with singleton pregnancies (OR 1.52, 95% CI 1.36–1.70).

### Distribution of GDM by socio-economic status

GDM rates varied by SES (Table 2) and were highest among women living in the most disadvantaged areas. A social gradient was evident, with adjusted odds of GDM decreasing with decreasing deprivation; incidence did not differ significantly between women in the middle two SES quartiles.

**Table 2:** Crude and adjusted incidence rates and incidence odds ratios of GDM by residential area socio-economic status, Victoria 1999–2008.

	INCIDENCE RATES (%)		INCIDENCE ODDS RATIOS (95% CI)					
SES Quartile	Crude	Age-adjusted	Crude	Adjusted for age group	Adjusted for age group and marital status	Adjusted for age group and plurality	Adjusted for age group and region of birth	Fully adjusted model**
1	4.86	5.52	REF	REF	REF	REF	REF	REF
(most deprived)		(5.33–5.71)						
2	4.18	4.45	0.86	0.8	0.79	0.79	0.93	0.93
		(4.29–4.61)	(0.81–0.90)	(0.75–0.84)	(0.75–0.83)	(0.75–0.84)	(0.88–0.98)	(0.88–0.98)
3	4.34	4.24	0.89	0.76	0.75	0.76	0.88	0.88
		(4.09–4.39)	(0.84–0.94)	(0.72–0.80)	(0.71–0.79)	(0.72–0.80)	(0.83–0.93)	(0.83–0.93)
4	4.12	3.62	0.84	0.64	0.64	0.64	0.73	0.73
(least deprived)		(3.49–3.75)	(0.80–0.89)	(0.61–0.68)	(0.60–0.67)	(0.61–0.68)	(0.69–0.77)	(0.69–0.77)
unknown	3.8	4.06	0.77	0.72	0.72	0.72	0.84	0.84
		(2.75–5.37)	(0.55–1.09)	(0.51–1.02)	(0.51–1.01)	(0.51–1.01)	(0.60–1.19)	(0.60–1.19)

** adjusted for age group, marital status, plurality, region of birth. Excludes those for whom SES quartile not known.

**Fig 1:**
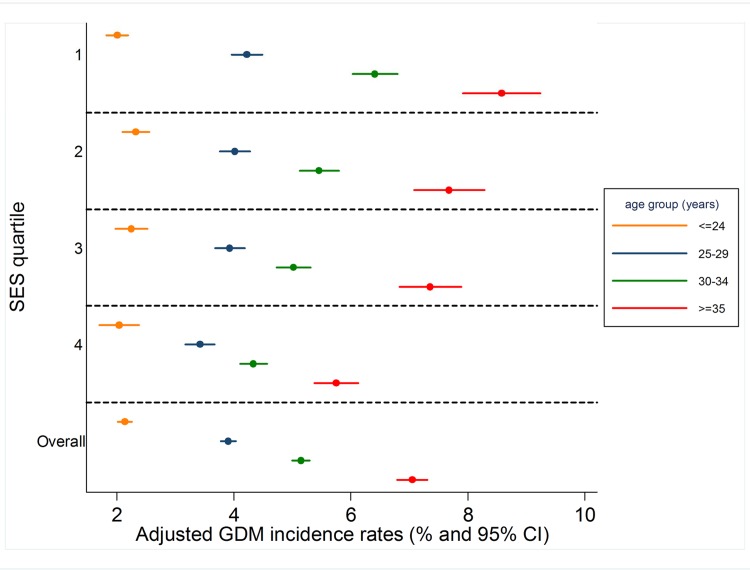
Adjusted GDM incidence rates by SES and maternal age at delivery, Victoria 1999–2008 Adjusted for marital status, plurality and maternal region of birth.

Fig. 1 presents adjusted GDM incidence rates stratified by SES and maternal age group. Within each SES stratum, odds of GDM increased with advancing maternal age. Within each maternal age group, SES associations varied – among younger women, GDM incidence was relatively stable across SES but a strong social gradient was apparent amongst older women. Patterns were similar for crude rates (data not shown).

### Distribution of GDM by maternal region of birth

GDM rates were highest for women born in the three Asian regions, even after age adjusting (Table 3). Crude odds of GDM were significantly higher in all migrant groups compared with non-Indigenous Australian-born women; adjusting for age had a pronounced effect for women born in North-West Europe and North-East Asia (the migrant groups with the oldest average age) and North Africa and the Middle East and Southern and Central Asia (the migrant groups with the youngest average age). For women born in North-West Europe, adjusting for age fully explained crude differences with the Australian-born non-Indigenous group. For all migrant groups, additionally adjusting for marital status, birth plurality and SES, either individually or collectively, had little effect.

**Table 3:** Incidence rates and incidence odds of GDM by maternal region of birth, Victoria 1999–2008.

	Incidence rates (%)		Incidence Odds Ratios (95% CI)					
REGION OF BIRTH	Crude	Age-adjusted	Crude	Adjusted for age	Adjusted for age, marital	Adjusted for age, plurality	Adjusted for age, SES	Fully adjusted*
								
Australia (non-Indigenous)	3.4	3.5	REF	REF	REF	REF	REF	REF
		(3.4–3.5)						
Oceania	4.3	4.4	1.26	1.28	1.28	1.28	1.28	1.28
		(3.9–4.9)	(1.11–1.43)	(1.13–1.45)	(1.13–1.46)	(1.13–1.46)	(1.13–1.45)	(1.13–1.45)
North-West Europe	4	3.4	1.16	0.97	0.97	0.97	0.99	0.99
		(3.0–3.7)	(1.04–1.29)	(0.87–1.08)	(0.87–1.08)	(0.87–1.08)	(0.89–1.10)	(0.89–1.10)
Southern & Eastern Europe	5.8	5.6	1.71	1.65	1.65	1.65	1.64	1.63
		(5.0–6.1)	(1.54–1.91)	(1.49–1.84)	(1.48–1.84)	(1.49–1.84)	(1.47–1.82)	(1.47–1.82)
North Africa & Middle East	5.1	6.2	1.51	1.86	1.86	1.86	1.8	1.79
		(5.5–7.0)	(1.33–1.71)	(1.64–2.11)	(1.64–2.11)	(1.64–2.12)	(1.58–2.04)	(1.57–2.03)
South-East Asia	9.2	9.3	2.85	2.88	2.88	2.89	2.78	2.79
		(8.8–9.7)	(2.69–3.02)	(2.71–3.06)	(2.71–3.06)	(2.73–3.07)	(2.62–2.95)	(2.63–2.96)
North-East Asia	11.2	10	3.52	3.13	3.12	3.14	3.2	3.2
		(9.3–10.7)	(3.25–3.81)	(2.89–3.39)	(2.88–3.39)	(2.90–3.41)	(2.95–3.47)	(2.95–3.47)
Southern & Central Asia	10.7	11.3	3.36	3.6	3.59	3.6	3.52	3.51
		(10.6–12.0)	(3.12–3.62)	(3.34–3.87)	(3.33–3.86)	(3.35–3.88)	(3.27–3.79)	(3.25–3.78)
Americas	4.5	4.1	1.33	1.2	1.2	1.21	1.22	1.23
		(3.5–4.8)	(1.12–1.56)	(1.02–1.42)	(1.02–1.42)	(1.02–1.42)	(1.04–1.45)	(1.04–1.45)
Sub-Saharan Africa	5.9	5.9	1.76	1.74	1.74	1.75	1.74	1.74
		(5.1–6.6)	(1.53–2.03)	(1.51–2.01)	(1.51–2.01)	(1.51–2.02)	(1.50–2.00)	(1.51–2.01)
Australia (Indigenous)	2.6	3.9	0.76	1.15	1.15	1.15	1.1	1.11
		(2.8–5.1)	(0.56–1.04)	(0.84–1.57)	(0.84–1.58)	(0.84–1.58)	(0.80–1.50)	(0.81–1.52)
								
Unknown	4.1	4.6	1.19	1.34	1.36	1.34	1.3	1.32
		(3.3–5.8)	(0.89–1.60)	(1.00–1.80)	(0.99–1.87)	(1.00–1.80)	(0.97–1.74)	(0.97–1.82)
								
Overall	4.4	4.4	n/a	n/a	n/a	n/a	n/a	n/a
		(4.3–4.4)						

*adjusted for age group, marital status, plurality and SES.

**Fig 2:**
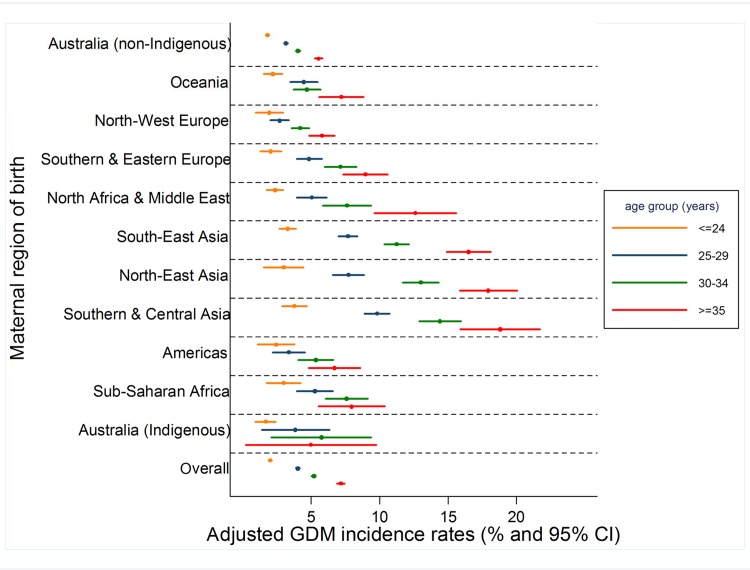
Adjusted GDM incidence rates by maternal region of birth and age at delivery, Victoria 1999–2008 adjusted for plurality, marital status and SES.

Fig. 2 presents adjusted GDM incidence rates stratified by maternal region of birth and age group. Differences in GDM rates were not pronounced among younger mothers, but became increasingly apparent amongst older women. Crude patterns were similar (data not shown).

## DISCUSSION

Pronounced socio-cultural differences in GDM burden exist among primiparous women in Victoria. These disparities vary by maternal age at delivery—among the youngest women, GDM rates were generally similar across socio-economic strata; however, amongst older women, a strong social gradient was evident. As reported by others [19], migrant and ethnic disparities were also particularly evident with advancing maternal age.

To our knowledge, this is the first study to report socio-economic gradients in GDM burden by maternal age and one of few that examines associations between area-based SES measures and GDM among pregnant women overall [7–9,12–14]. Such associations were last reported for Victoria for 1996 [13]. At that time, adjusted GDM incidence differed only between the most and least deprived women. Conversely, in our study for the decade to 2008, a strong overall social gradient existed for primiparous mothers after multivariate adjustment (Table 2) and this was due to a social gradient predominantly amongst older women (Fig. 1). There are some methodological differences between the two studies, but these are unlikely to account for the emergent gradient. Notably, maternal age structure has changed – mean age at delivery among primiparous women in Victoria increased from 27.2 years in 1995 to 29.1 years in 2008; women aged over 35 years comprised 14.6% of all women giving birth in Victoria in 1995, and 26.5% in 2008 [20].

Associations between maternal age and GDM are well documented (for example, [12–14,19,21]). This study adds to the literature by demonstrating that older women living in less deprived areas have considerably lower GDM rates than older mothers living in more deprived areas; it would seem that some factor embedded within the SES construct may modulate the established association between GDM and advancing maternal age. Alternatively, as GDM rates did not vary markedly by SES among younger women, it may be that younger age is protective against neighbourhood characteristics that confer risk. It is not possible to infer causation or directionality, or to discern the individual SES components contributing to this gradient. Some components of the neighbourhood SES construct are potentially modifiable. Therefore, future research should seek to identify the specific aspects of residential area SES that are associated with GDM risk. This work has important implications: efforts should be directed to reduce the risk amongst all older women to that of the least deprived older group.

The finding that GDM complicated 4.4% of primiparous pregnancies in Victoria overall during the study period is in the range expected, and consistent with national reports that GDM was diagnosed in 4.6% of all pregnancies among women aged 15–49 years in 2005–2006 [22].

Our findings are also consistent with reports from New South Wales, Australia of an inverse association between area-based SES and GDM burden for the period 1995 to 2005 [12]. However, independent associations between area-based SES measures and GDM are not reported universally [8,9].

Migrant differences in GDM burden are well established and our results are consistent with Australian findings from the last four decades [12,13,19,21–26]. Our finding of no significant difference in GDM burden between Australian-born Indigenous and non-Indigenous women differs from national reports [22] and may be due to the small number of Indigenous women rendering this study underpowered to detect any such effects.

Migrant disparities in GDM in Victoria are particularly pronounced amongst older mothers [19]. In our study, even after considering where a woman was born, amongst older mothers, where she lives was still strongly associated with risk of GDM. Similarly, particularly among the oldest women, ethnic differences persisted even after SES adjustment. Together, these results would suggest that with advancing maternal age, both where a woman was born and where she lives independently influence GDM risk.

Some of this variation may be due to residual confounding by factors not considered in this study, in particular by socio-cultural differences in BMI. As maternal BMI data were not available in the VPDC over the study period, BMI effects on GDM rates within and between socio-economic and migrant/ethnic groups could not be examined. Few studies have addressed associations between BMI, SES and GDM [27]. Data on diet and physical activity patterns were also unavailable and so the influence of such factors could not be considered. Future work should examine the extent to which socio-cultural disparities in GDM burden are explained by socio-cultural disparities in obesity and other risk factors.

### Strengths and limitations

There are several strengths to this work. The data source comprised a comprehensive perinatal database that captures virtually all births in the state. Birth report forms are completed by attending clinicians at the time of delivery, meaning GDM ascertainment is not dependent on maternal recall. Victorian birth report forms were consistent over the study period, as were GDM screening criteria and diagnostic thresholds. This is one of few studies to consider only women in their first pregnancy. Restricting our focus to primiparous women enabled examination of socio-cultural associations with GDM independent of the effects of parity, interpregnancy weight gain and GDM recurrence in subsequent pregnancies. It also means that women are only represented in the data set once. This ensures statistical rigour, as regression analyses assume that observations are independent – socio-cultural differences in screening rates may influence such assumptions are violated if the same woman is included in a data-set multiple times, as may occur when the study base comprises all pregnancies over a given time period, during which a woman may have given birth more than once.

There are some limitations. The region of birth classification used was broad. The composite residential area SES measure is based on 2006 census data and this study assumes that the SES score for a given postcode was consistent over the study period. Use of an area-level construct renders this work susceptible to the ecological fallacy, if an individual woman’s SES differs from that assigned based on her residential area. Individual-level SES data were not available. There are discrepant reports in the literature about associations between GDM and some individual-level SES measures, such as income [6,10] and education [4,5,26].

Our findings are not necessarily generalisable to multiparous women. For example, family size may influence choice of residential area and thus the neighbourhood SES score assigned. It was not possible to ascertain if women were screened for GDM – socio-cultural differences in screening rates may influence case ascertainment and estimates of GDM rates. The high GDM burden among low SES women and in some migrant groups may represent pre-existing but undiagnosed diabetes management guidelines as postpartum oral glucose tolerance test results were not available, this cannot be quantified in our data set. Finally, it is possible that some women may have had a prior pregnancy that was not carried to at least 20 weeks and therefore have been included in this study due to being primiparous despite being multigravid. It has been reported that in predominantly primiparous women in China, a history of spontaneous abortion was significantly associated with increased GDM risk [28]. We did not consider history of terminations or spontaneous abortions, so it is unclear to what extent this may have influenced our results.

### Conclusions

That ethnic disparities are particularly pronounced amongst older mothers has been previously documented. This study demonstrates that in addition to older Asian mothers, older women living in deprived areas are at greatest risk of GDM, but socio-economic variation is not particularly evident among the youngest first-time mothers. As the average age at first delivery continues to increase [20] and the proportion of older mothers grows, there is an urgent need to identify and address the elements of the area-based SES construct that are associated with this increased risk. This is especially important because some such factors may be modifiable. Given the health risks to the infant of a GDM pregnancy, if GDM and its sequelae are disproportionately high among women in deprived areas, the future burden of obesity and type 2 diabetes may also be concentrated in disadvantaged groups. Addressing the disparity is a matter of intergenerational equity: the intrauterine environment confers increased risk to a child. We cannot allow neighbourhood environments to magnify this risk.
